# Spatial analysis and influencing factors of pulmonary tuberculosis among students in Nanning, during 2012–2018

**DOI:** 10.1371/journal.pone.0268472

**Published:** 2022-05-24

**Authors:** Dan-ling Yang, Wen Li, Meng-hua Pan, Hai-xia Su, Yan-ning Li, Meng-ying Tang, Xiao-kun Song

**Affiliations:** 1 Department of Health Statistics, School of Public Health, Guangxi Medical University, Nanning, Guangxi, China; 2 School of Information and Management, Guangxi Medical University, Nanning, Guangxi, China; Shandong Public Health Clinical Center: Shandong Provincial Chest Hospital, CHINA

## Abstract

**Background:**

Economically underdeveloped areas in western China are hotspots of tuberculosis, especially among students. However, the related spatial and temporal patterns and influencing factors are still unclear and there are few studies to analyze the causes of pulmonary tuberculosis in students from the perspective of space.

**Methods:**

We collected data regarding the reported incidence of pulmonary tuberculosis (PTB) among students at township level in Nanning, from 2012 to 2018. The reported incidence of pulmonary tuberculosis among students in Nanning was analyzed using spatial autocorrelation and spatial scan statistical analysis to depict hotspots of PTB incidence and spatial and temporal clustering. Spatial panel data of the reported incidence rates and influencing factors at district and county levels in Nanning were collected from 2015 to 2018. Then, we analyzed the spatial effects of incidence and influencing factors using the spatial Durbin model to explore the mechanism of each influencing factor in areas with high disease prevalence under spatial effects.

**Results:**

From 2012 to 2018, 1609 cases of PTB were reported among students in Nanning, with an average annual reported incidence rate of 14.84/100,000. Through the Joinpoint regression model, We observed a steady trend in the percentage of cases reported each year (P>0.05). There was spatial autocorrelation between the annual reported incidence and the seven-years average reported incidence from 2012 to 2018. The high-incidence area was distributed in the junction of six urban areas and spread to the periphery, with the junction at the center. The population of college students, per capita financial expenditure on health, per capita gross domestic product, and the number of health technicians per 1,000 population were all influencing factors in the reported incidence of PTB among students.

**Conclusion:**

We identified spatial clustering of the reported incidence of PTB among students in Nanning, mainly located in the urban center and its surrounding areas. The clustering gradually decreased from the urban center to the surrounding areas. Spatial effects influenced the reported incidence of PTB. The population density of college students, per capita health financial expenditure, gross domestic product (GDP) per capita, and the number of health technicians per 1,000 were all influencing factors in the reported incidence of PTB among students.

## Background

Pulmonary tuberculosis (PTB) is an airborne disease caused by the pathogen *Mycobacterium tuberculosis*. PTB is the first cause of death by an infectious agent until the emergence of COVID-19 [1]. According to the World Health Organization, there were an estimated 10 million new cases of tuberculosis (TB) in 2019, with 1.41 million deaths. China accounted for 8.4% of total cases worldwide, ranking third globally [2].

China has been undergoing a process of epidemiological transition, with an increasing number of chronic and non-transmissible diseases, but infectious and parasitic diseases are decreasing. However, PTB remains a significant public health problem in China [3], especially in areas that are relatively poor and where residents experience social exclusion [4]. Guangxi is not only an economically underdeveloped autonomous region in southern China, it also has a large number of individuals with TB [5]. The National Notifiable Disease Reporting System (NNDRS) states that approximately 50,000 new TB cases are reported in Guangxi every year. The province has the fifth-highest TB burden in China, with annual reports showing that about 100 of every 100,000 people in Guangxi have TB [6].

In recent years, the number of PTB cases has increased rapidly, leading to outbreaks in schools [7]. Hence, the reported incidence of PTB among students has shown an upward trend [8]. School is one of the densely populated public places [9], where PTB can easily spread via airborne transmission, which can lead to epidemics in the school and nearby areas [10]. TB outbreaks have a strong impact on society, especially on students and their families. Once TB begins to spread widely among students, panic can affect the local community and the whole of society [5, 11]. The reported incidence of PTB among students in Nanning is continually rising [12]. However, few studies exploring the spatial epidemiology of PTB among students have been conducted in Nanning. The foundation of public health in western China is still fragile, and medical treatment capacity remains insufficient [13]. Therefore, TB outbreaks among students lead to considerable challenges. We analyzed the incidence of PTB among students in economically underdeveloped areas of China and identified high-risk areas and important influencing factors. This is of great importance for protecting people’s health in disadvantaged areas [14, 15].

In this study, we aimed to determine the epidemiological spatial factors associated with an increased risk of TB among students in Nanning city, Guangxi Province, from 2012 to 2018. We also aimed to identify possible concentrated areas and the clustering time of TB using geographic information system (GIS) “hotspot” analysis.

## Methods

### Study setting

Nanning has a population of about 8.74 million. The province covers an area of 22,100 square km and has a 450-km border with Vietnam. There are 12 counties and districts in Nanning [16].

### Data source

We extracted data of students included in PTB surveillance from January 2012 to December 2018 from the NNDRS, which was established and is operated by the Chinese Center for Disease Control and Prevention (CCDC). We obtained PTB data from the Nanning Institute of Tuberculosis Control and Prevention with the permission of the CCDC.

Each case report contains basic demographic information and clinical diagnostic information. We analyzed cases by county and mapped them by area code. Annual population the estimates of each administrative district from 2012 to 2018 were obtained from the Chinese County Statistical Yearbook and Nanning Statistical Yearbook. We constructed a panel data frame with six indications from the Guangxi Statistical Yearbook and Nanning Statistical Yearbook. We obtained vector map files from the Resource and Environment Science and Data Center.

### Time trend analysis

We performed data analysis using joinpoint regression model to evaluate the monthly time trend of students suffering from tuberculosis. The incidence of TB was taken as the dependent variable and the epidemic month as the independent variable. The Monte Carlo permutation test selected the best model for inflection points, applying 999 permutations and considering the highest residue determination coefficient (R^2^).

To describe and quantify the time trends, we calculated the Monthly Percent Change (MPC) [17] and their respective confidence intervals (95% CI). Once more than one significant inflection was detected during the study period, the average monthly percentage changes (AMPC) were calculated. Time trends were considered statistically significant when MPC or AMPC had a P-value <0.05, and their 95% CI did not include a zero value. A positive and significant MPC or AMPC value indicates an increasing trend; a negative and significant MPC or AMPC indicates a decreasing trend; non-significant trends are described as stable [18].

### Spatial autocorrelation analysis

Our study used the Global Moran’s I statistic to analyze the spatial autocorrelation of infection prevalence (based on feature locations and attribute values) to evaluate the overall data pattern and trend. The spatial correlation shown in the Local Moran’s I index provides us with local indicators of spatial correlation (LISA), which can be used to detect regions with significant spatial correlation and clustering measures [19].

### Spatiotemporal scan statistic

Spatial scanning statistics is a tool for detecting disease clusters in space and time. The circular spatial scan statistic and SaTScan software proposed by Kulldorff have been used widely in various epidemiological studies and disease surveillance [20]. SaTScan can be applied for both temporal and spatial factors to scan different regions with a dynamic circular window and detect the spatiotemporal aggregation of diseases, which is a beneficial supplement to simple spatial scanning analysis. Flexible spatial scan statistics can detect the clustering of arbitrary shapes that circular spatial scanning statistics cannot detect. Owing to the more flexible shape of the scanning window, FleXScan can detect spatial clustering areas closer to the actual situation, with a higher application value [21]. In this paper, we used SaTScan to supplement time and information. In contrast, the FleXScan scan was used to provide highly accurate supplemental data, which can be combined to detect the spatial aggregation of PTB, resulting in better reflection of the disease distribution.

### Spatial Durbin model

The spatial Durbin model (SDM) is a common type of spatial lag model (SLM), spatial lag of X model, and spatial error model (SEM). The SDM can be used to investigate not only the influence of local variables on dependent variables but also the influence of adjacent regional dependent variables and their independent variables [22, 23]. Its basic formula is:

$$y_{it}=\rho \;W_{1}y_{it}+\beta \;X_{it}+\theta \;W_{2}X_{it}+\varepsilon_{it}$$

$$\varepsilon_{ab}∼N(0,\sigma^{2}I_{n})$$

y_it_ is the dependent variable of district i in year t (the time dimension is 2015–2018); W_1_ corresponds to the spatial weight matrix; ρ is the spatial parameter of interest, which reflects the endogenous spatial interaction between district i and adjacent districts; β is the vector of explanatory variable coefficient; X_it_ is the independent variable affecting the incidence of PTB in i district and t years. θ reflects exogenous interaction and adds the values of explanatory variables of neighboring districts to the set of conventional explanatory variables, on average; the matrix W_2_X_it_ represents the spatial lag effect related to explanatory variables. ε represents an error term, which is not related to the cross-country and time-varying explanatory variables, assuming that it is typically distributed.

The model based on spatial panel data can also be divided into a fixed effects model and random effects model. Regarding the choice of these two models, if the data happened to be randomly sampled for the population, the random effects model should be adopted. If the data sample includes nearly the entire population, the fixed effects model is more appropriate [24]. The objective of this study was to investigate 12 districts and their counties in Nanning. The 12 districts and counties form a whole spatial objective, which cannot be randomly sampled, so we used the fixed effects model.

In general, after estimating the spatial regression parameters, the SDM should also be used to calculate direct effects and indirect effects. The measurement of spatial effects is conducted by dividing it these into a “direct effect” and an “indirect effect.” The direct effect is the influence of the change in the local independent variable on the local dependent variable, and the indirect effect is the influence of the change in the local independent variable on the adjacent region [22]. Therefore, a dependent variable of a region is affected by both the local independent variable and the adjacent independent variable.

The applicability of SDM was tested to see if the SDM can be simplified to an SLM or SEM. The LR test and Wald test of the SDM showed the following: Wald_spatial_lag = 44.16, P<0.001; LR_spatial_lag = 18.45, P<0.05;

Wald_spatial_error = 40.78, P<0.001; and LR_spatial_error = 17.54, P<0.05.

Therefore, the SDM cannot be reduced to an SLM or SEM. In addition, the results show that the W * X coefficient is significant, which avoids the lack of variables, indicating that it is reasonable for us to choose the SDM with fixed effect.

To eliminate the influence of heteroscedasticity, we performed logarithmic processing for all variables.

## Results

### Descriptive analysis of PTB cases

**Fig 1:**
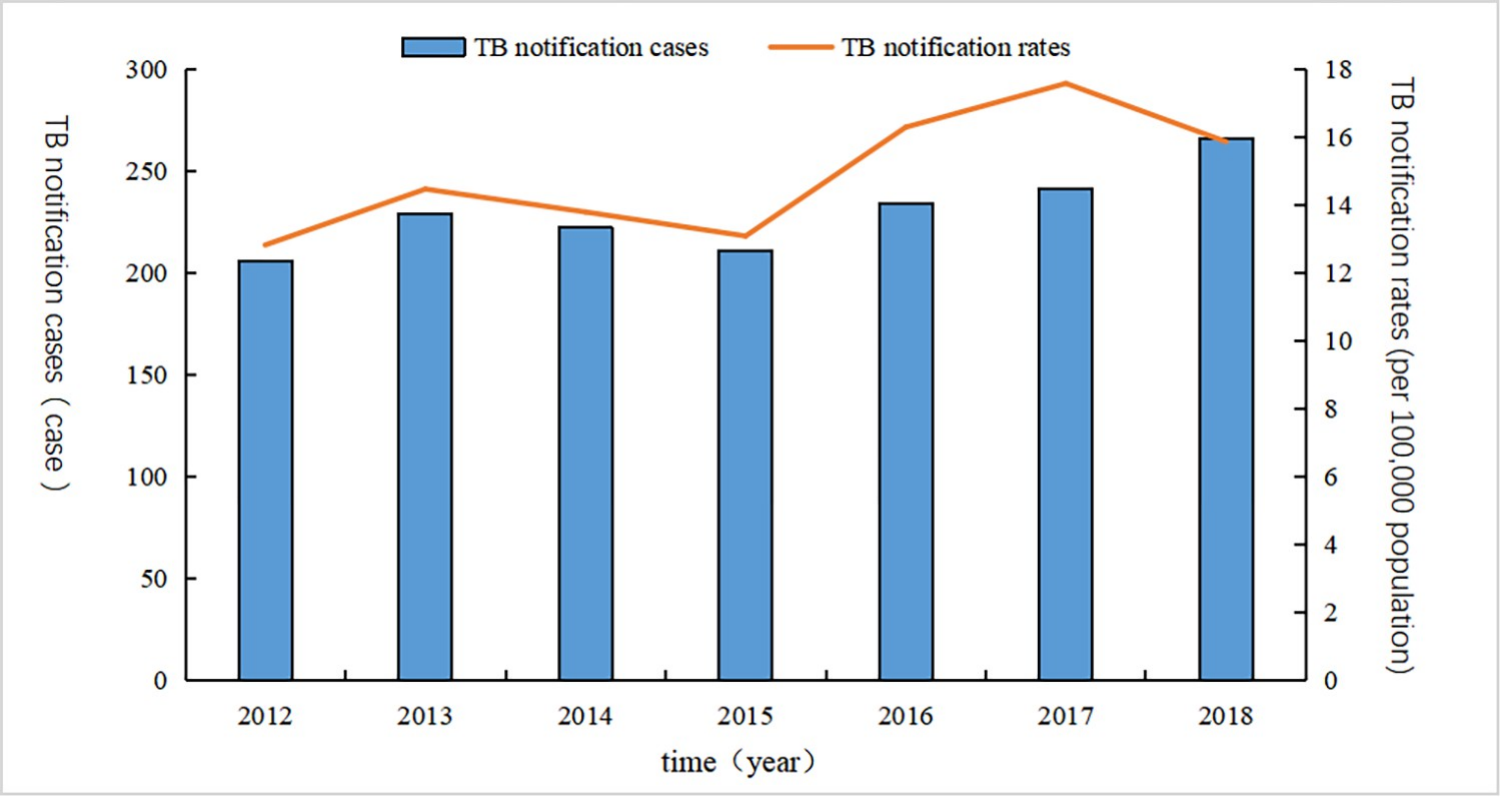
Reported incidence of pulmonary tuberculosis among students in Nanning, from 2012 to 2018.

From 2012 to 2018, a total of 1609 TB cases among students were reported in Nanning. Fig 1 shows the average annual detection rate for all PTB cases was 14.84 cases per 100,000 population. The total reporting rate of TB cases increased significantly from 12.82 per 100,000 people in 2012 to 17.57 per 100,000 people in 2017 ($\chi_{trend}^{2}=11.37,P=0.01$).

**Fig 2:**
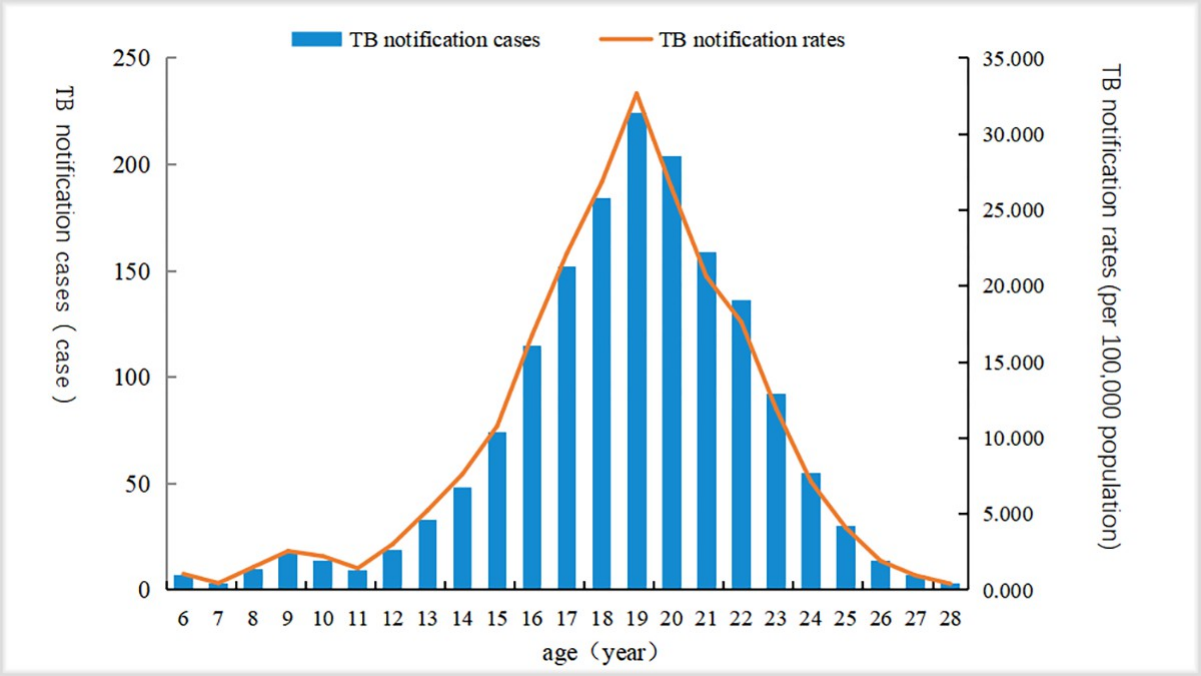
Overall age distribution of reported cases of pulmonary tuberculosis among students in Nanning, from 2012 to 2018.

The reported age of students with PTB in Nanning was between 6 and 28 years old, with an average age of 18.77 ±3.51 years old, mainly between 16 and 22 years of age. In this age group, 1174 cases were notified, accounting for 72.96% of the total reported cases. The notification rate of PTB was highest among students under 19 years old. The demographic characteristics of PTB cases among students in Nanning from 2012 to 2018 are shown in Fig 2.

**Fig 3:**
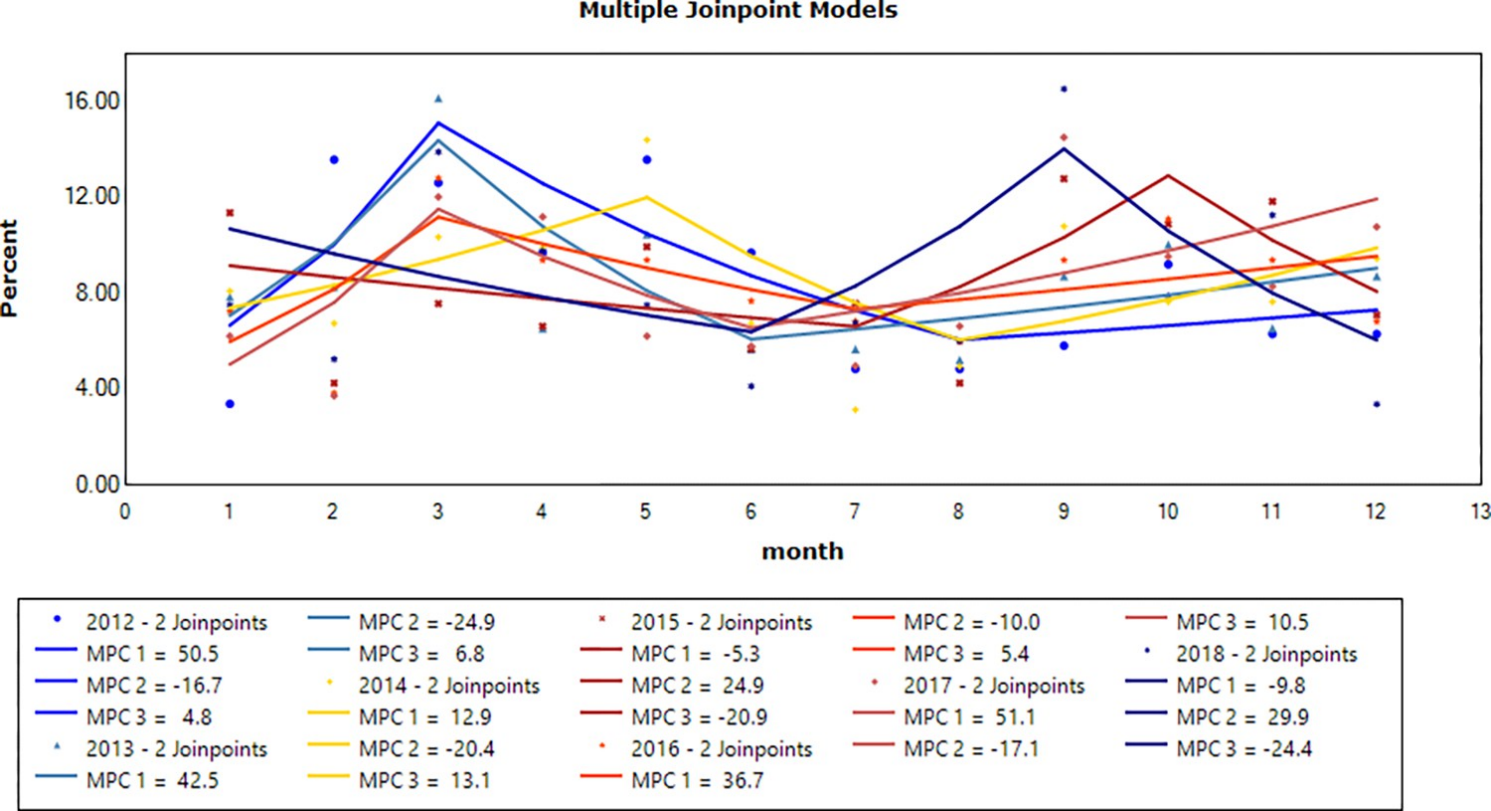
Temporal distribution of reported cases of pulmonary tuberculosis cases by year among students in Nanning, from 2012 to 2018.

A clear temporal trend variation of PTB cases among students in Nanning from 2012 to 2018 can be seen in Fig 3. Through the Joinpoint regression model, we observed a steady trend in the percentage of cases reported each year (P>0.05). However, the peak of tuberculosis cases was almost in March, May, or September, and there was a downward trend from January to February or from June to August.

### Spatial autocorrelation analysis

Global spatial autocorrelation analysis of the annual incidence of TB among students in Nanning from 2012 to 2018 showed that the annual Moran’s I was significant (Table 1), indicating the distribution of TB in Nanning had spatial autocorrelation. Because annual Moran’s I statistics was between 0.216 and 0.273 (*P*<0.05), the analysis indicated a positive spatial correlation between the high–high cluster and low–low cluster in the spatial distribution.

**Table 1:** Global spatial autocorrelation analyses for annual TB notification rate among students in Nanning, from 2012 to 2018.

year	Moran’s I	Z	*P* value
2012	0.239	4.652	0.017
2013	0.216	4.219	0.021
2014	0.224	4.373	0.017
2015	0.228	4.450	0.018
2016	0.254	4.706	0.011
2017	0.264	4.861	0.008
2018	0.273	5.072	0.006

**Fig 4:**
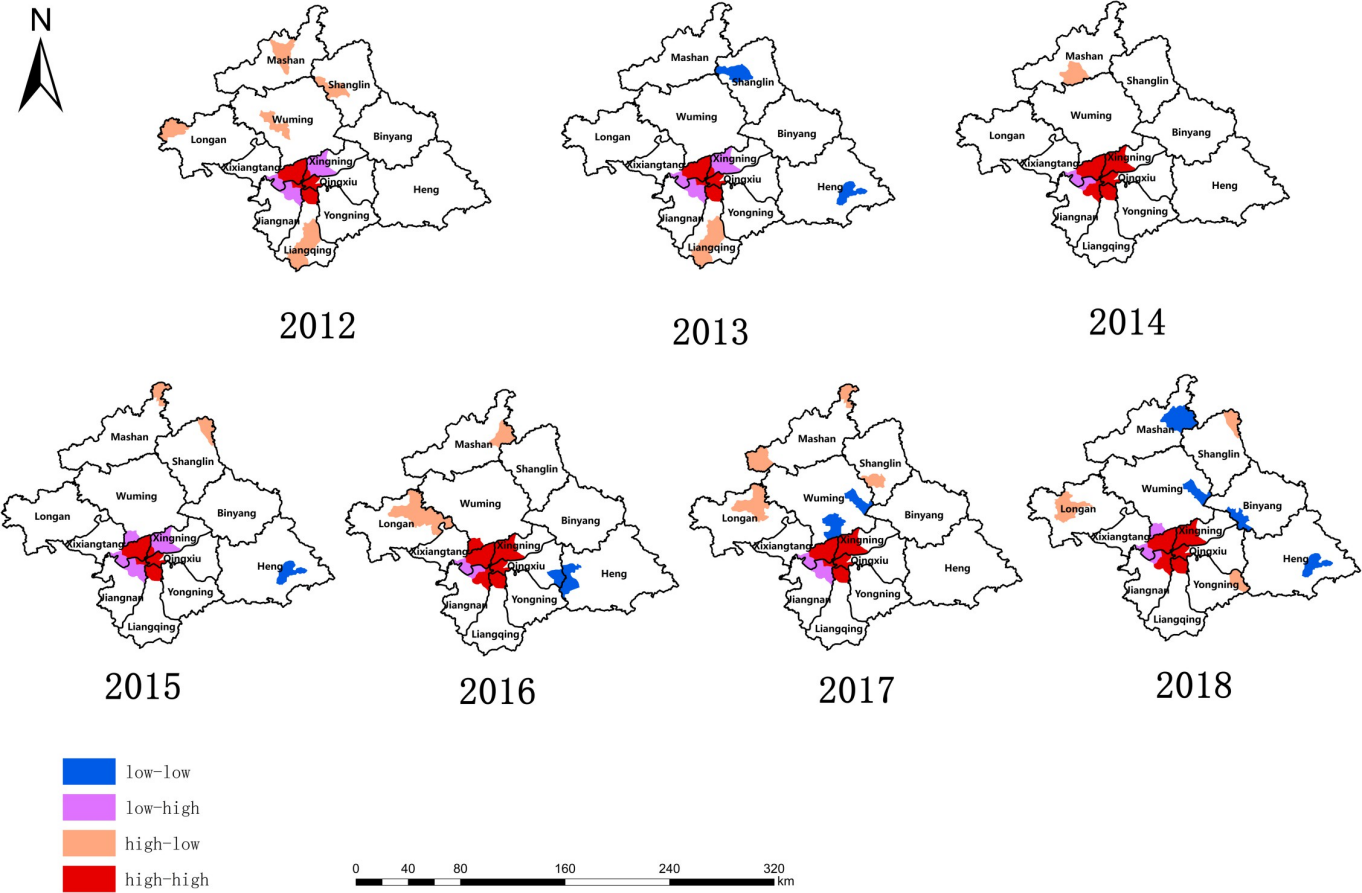
LISA spatial clustering pattern of reported incidence of pulmonary tuberculosis among students in Nanning from 2012 to 2018.

Fig 4 shows the analysis results of local spatial autocorrelation. According to the survey, hotspots of PTB among students in Nanning from 2012 to 2018 were concentrated in Chaoyang Street, Jianzheng Street, Fujianyuan Street, and Huaqiang Street. There were many small hotspot areas that changed dynamically with time. For example, Jiangnan Street, Nahong Street, Dashatian Street, and Santang town were always hotspots for PTB. However, there was always a slight change in the TB hotspots in Nanning each year. The accumulation of cold spots is not significant, among which Hengzhou Town of Hengxian County and Jiafang Township of Mashan County are the most obvious cold spots for TB in Nanning.

### Spatiotemporal clustering analysis by SaTScan

**Fig 5:**
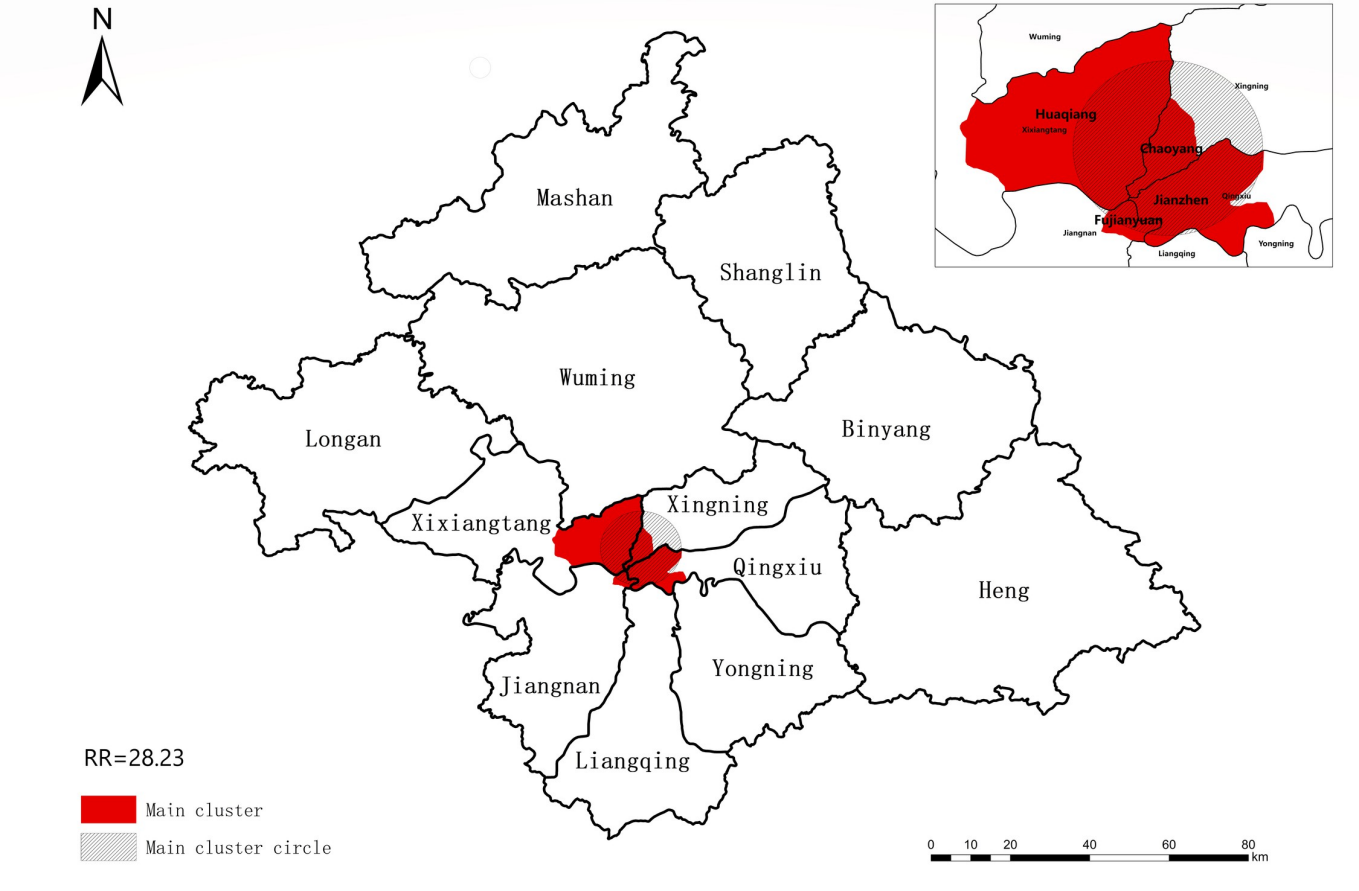
SaTScan for overall reported incidence of pulmonary tuberculosis among students in Nanning from 2012 to 2018.

We conducted spatiotemporal scans of the reported rate of PTB cases among students in Nanning from 2012 to 2018. Our results showed that the reporting rate of TB cases was spatially distributed and clustered (Fig 5). The clustering time was from January 2012 to December 2015. The most likely clustered region was mainly distributed in the middle of Nanning, covering Chaoyang Street, Jianzheng Street, Fujianyuan Street, and Huaqiang Street (LLR = 1119.90,RR = 28.23,P<0.001).

### Comprehensive spatial distribution of PTB reports

**Fig 6:**
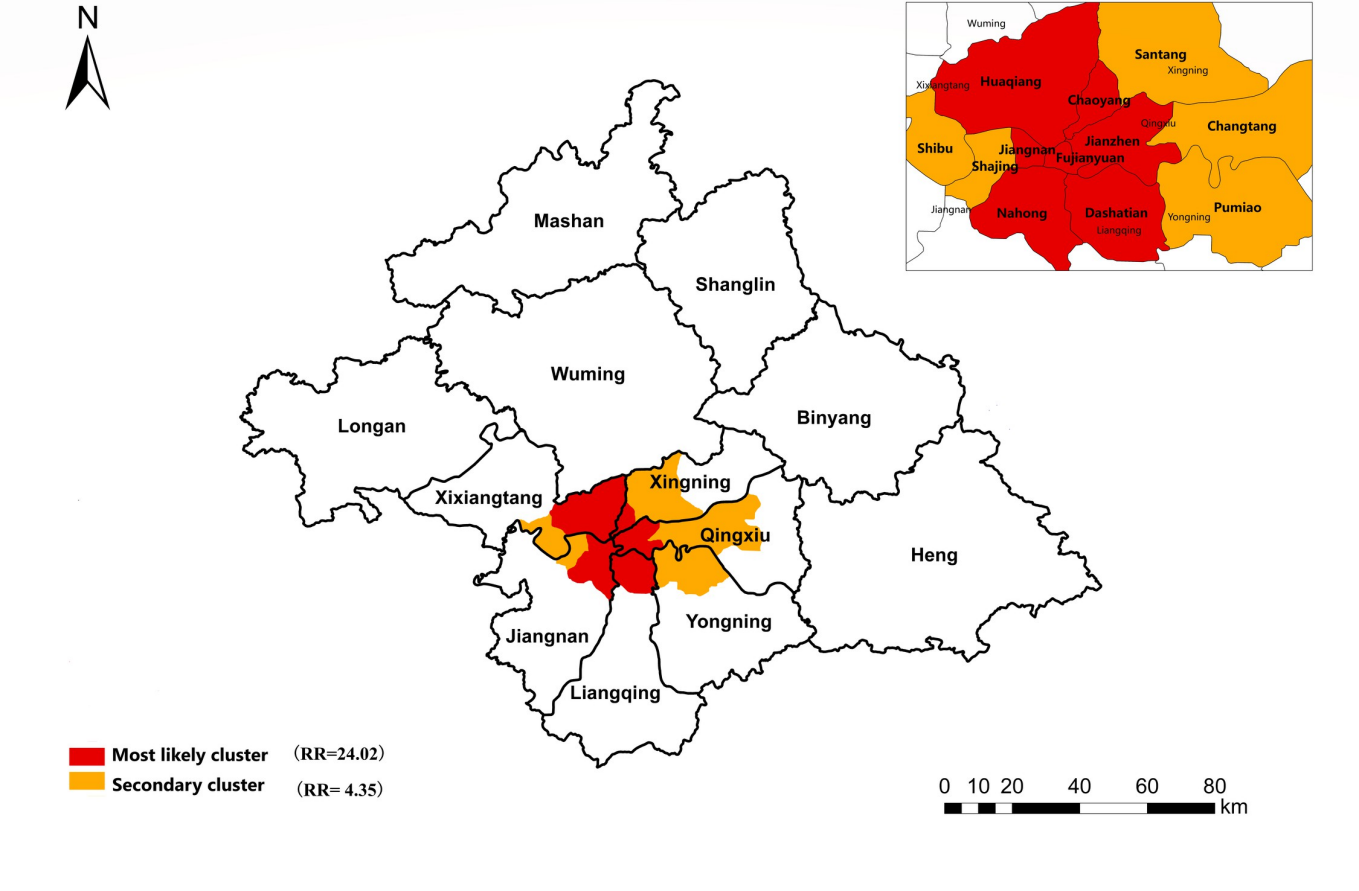
High-incidence clustering of reported incidence of pulmonary tuberculosis among students in Nanning.

Global autocorrelation analysis, LISA, SaTScan, and FleXScan were used to map correlations of the spatial distribution and to detect the space–time clusters of PTB cases among students in Nanning during 2012–2018 (Fig 6). In terms of spatial structure, the high-risk areas for PTB in Nanning were mainly located at the junction of Xingning District, Qingxiu District, Xixiangtang District, Liangqing District, and Xining District. Spatial autocorrelation with spatial scan statistical analysis showed roughly the same results.

The high-risk areas of PTB cases among the students in Nanning could be divided into the most likely cluster and secondary cluster regions. The most likely cluster region comprised Chaoyang Street, Jianzheng Street, Fujianyuan Street, Huaqiang Street, Jiangnan Street, Nahong Street, and Dashatian Street (LLR = 266.84,RR = 24.02,P<0.001). Secondary cluster regions were Shajing Street, Shibu Street, Santang Town, Changtang Town, and Pumiao Town (LLR = 13.86,RR = 4.35,P<0.001).

### Spatial econometric model

As a result, six variables were included in the SDM: demographic variables (population of college students, middle school students, and primary school students), economic variables (GDP per person, health financial expenditure per person), and health resources (number of health technicians per 1000 persons). The results of this analysis are shown in Table 2.

**Table 2:** Spatial Durbin model estimation results.

Variables	Coefficient	P value	Variables	Coefficient	*P* value
Per capita GDP	-0.080	0.823	W×Per capita GDP	1.792**	0.026
College students’ population density	0.263***	0.004	W×College students’ population density	-0.202	0.360
Middle school students’ population density	-0.321	0.126	W×Middle school students’ population density	-0.515	0.445
Primary school students’ population density	-0.020	0.924	W×Primary school student	0.584	0.477
Per capita health financial expenditure	-0.417**	0.034	W×Per capita health financial expenditure	0.262	0.750
The number of health technicians per thousand	0.278	0.051	W×The number of health technicians per thousand	-1.007**	0.043
ρ (spatial self-correlation coefficient)	-2.70***	0.007			

Notes

*** and ** denote statistical significance at the 1% and 5% significance levels, respectively.

The following table is the same: W represents the spatial weight matrix.

The results of estimation using the SDM showed that the spatial model coefficient (ρ) was significant (Table 2). Thus, the spatial spillover effects of the notification rates of TB cases among students in Nanning were highly significant. The population density of college students had a significantly positive correlation with the notification rates of TB cases. In contrast, the health financial expenditure per person had a significantly negative correlation with the notification rates of PTB cases. Among spatial item coefficients, only W × per capita GDP and W × number of health technicians per 1000 showed a significant correlation with notification rates of PTB, and others were not significantly associated with notification rates of PTB.

SDM involves the spatial lag terms of dependent and independent variables. Hence, the total effects of independent variables on dependent variables were decomposed into direct and indirect effects via the effect decomposition technique.

First, in the direct effect, the results showed that population density of college students and health financial expenditure per person positively impacted the notification rates of PTB in the local region. As for the indirect effect, GDP per person positively impacted the notification rates of PTB in neighboring regions. Second, Table 3 shows that the direct and indirect effects of the number of health technicians per 1000 are statistically significant, indicating that the number of health technicians per 1000 population has a significant impact on the incidence of TB in the local and surrounding areas.

**Table 3:** Estimation results of the spatial effect decomposition of the Spatial Durbin mode.

Variables	Direct Effect	*P* value	Indirect Effect	*P* value
per capita GDP	-0.259	0.432	1.493**	0.010
College students’ population density	0.290***	0.001	-0.268	0.088
Middle school students’ population density	-0.276	0.258	-0.324	0.591
Primary school students’ population density	-0.085	0.713	0.538	0.426
Per capita health financial expenditure	-0.468***	0.002	0.341	0.598
The number of health technicians per thousand	0.407**	0.010	-0.935**	0.025

## Discussion

This study revealed the temporal, spatial, and temporal distributions of TB prevalence among students in southwest Nanning from 2012 to 2018. We used SaTScan and FleXScan to supplement the relevant spatiotemporal information. Finally, using the population density of college students and other influencing factors in Nanning from 2015 to 2018 to construct panel data, we used the SDM to investigate the relationship between influencing factors and disease incidence.

The notification rate of TB among students in Nanning increased during the 8-year study period. The incidence of TB increased from 12.82/100,000 population to 17.57/100,000 population. This result is consistent with that of other researchers [25, 26]. However, the prevalence rate of TB in the total population of Nanning has decreased across the same period [27, 28], indicating that the proportion of reported cases of TB among students in the overall population may have increased. This is mainly because the Guangxi government and health administrative departments have paid more attention to the control and incidence rate of TB among students in recent years. Nanning has responded positively and continues their work. The number of reported cases increased continuously between 2012 and 2018, probably because the proportion of incident cases detected increased over that period.

In the present study, cases were mainly among individuals aged 16–22 years, that is, mainly high school or college students. This may be because most senior high school students have high levels academic pressure for college entrance examinations and therefore more vulnerable to infections [11, 29]. The environment can also influence the occurrence of TB [30]. Most boarding students’ quarters would be crowded and have little air circulating. In addition, students might conceal illness for fear of being required to take time off from school, which can lead to the rapid spread of TB. Finally, the physical examination that is part of college entrance examinations increases the detection rate of TB among senior high school students and college students.

The results show no obvious periodic change trend in the prevalence rate of TB among students in Nanning. However, the peak period is in March, May, or September. This result corresponded to the operating time of the school and is similar to other studies [31, 32]. The reason may be that the school year begins between March and May, and students return to campus life, where it is easy to contact pathogens that cause infection. The second peak of TB is mainly at the beginning of the autumn semester in September. With the rapid development of prevention and control, the epidemic situation will disappear rapidly, so there is no obvious long-term change trend.

The distribution of the notification rate of TB among students in Nanning is the same as that of the whole population [27, 33]. The locations of high incidence are mainly concentrated in densely populated areas or a location with many schools.

Xingning District, Qingxiu District, and Xixiangtan. Districts were all very high-risk areas in terms of reported cases and incidence. Our results showed that the high incidence of TB among students in Nanning was located in an urban center with high-quality educational resources, medical institutions with advanced facilities, and convenient transportation facilities. The results of this study are similar to those of previous studies [16, 34]. Those studies found a significant difference between the spatial distribution of PTB among students and that of the whole population in Guangxi. The spatial aggregation of the incidence of PTB in Guangxi is mainly located in economically disadvantaged areas. However, the spatial aggregation of TB among students was located in the center of economically developed urban areas.

The results of SDM show the following: First, the population density of college students has a significantly positive effect on the reported incidence. College students are from all over the country, so this group has high mobility that is not easy to control. Second, per capita health expenditure has a negative impact on the reported incidence. This is similar to other research [35, 36]. Health expenditure is mainly used for public health and the improvement of the health of the local population, including the costs of TB treatment. High-incidence areas bear a heavy disease burden, and the population inflow from surrounding areas aggravates the shortage of local health resources. Therefore, reducing local per-capita health expenditure is not conducive to disease prevention and control.

We found that GDP per person indirectly affects the reported incidence of TB among students [37, 38]. This shows that the level of health economic development in this area will not only affect the local reported incidence but also have a positive impact on neighboring areas. Our study population was students, who do not represent an independent group in society. This population is great influenced by their family and school. In addition, economic factors do not directly affect this group through income but can be seen in the comprehensive effect of economic development, spatial location, and population mobility.

The number of health technicians per 1000 people has both direct and indirect effects. Usually, the distribution of health resources has a certain relationship with the local economy [39], and most health resource facilities are concentrated in economically developed areas [40]. Nanning is an economically developed region of Guangxi Province, and compared with rural centers, urban centers have a large number of high-quality medical resources. The floating population in urban areas has dramatically increased the risk of infection among local students, resulting in an increased number of TB cases. Adequate human resources have a positive role in TB screening, increasing the number of screening cases [41]. This is because of the direct effect of surrounding areas, which not only lack medical resources but also affect disease sources and healthy human resources, influencing local disease screening and reducing the reported incidence [38]; This is a manifestation of indirect effect.

Our research had some limitations. First, because some variables could not be obtained, only four years of data for influencing factors, from 2015 to 2018, were extracted and analyzed. Second, it is difficult to quantify the effectiveness of policies at the township or district level, so our findings failed to reflect the impact of policy implementation on changes in the reporting of TB outbreaks in various regions. In future research, we will collect more data for analysis.

### Conclusions

This study identified the spatiotemporal aggregation and influencing factors of TB cases among students in Nanning at the county level from 2012 to 2018. First of all, the most likely clustering areas are the center region of Nanning. Therefore, these areas should be priorities for TB control. Second, the reported incidence of PTB among students in Nanning was affected by spatial effects. The population density of college students, per capita health expenditure, per capita GDP, and the number of health technicians per 1000 population were found to be influencing factors.

## Supporting information

S1 Fig (PNG)

S2 Fig (PNG)

S1 Table (DOCX)

## Abbreviations

PTB: pulmonary tuberculosis
MTB: Mycobacterium tuberculosis
WHO: World Health Organization
TB: tuberculosis
NNDRS: National Notifiable Disease Reported System
CCDC: Chinese Center for Disease Control and Prevention
LISA: local index of spatial correlation
SDM: Spatial Durbin Model
SLM: Spatial lag Model
SLX: Spatial lag of X model
SEM: Spatial Error Model
GDP: Gross Domestic Product
