# Supplementary figures and images for: Spatial analysis and influencing factors of pulmonary tuberculosis among students in Nanning, during 2012–2018
Source: PLoS One. 2022 May 24;17(5):e0268472. doi: 10.1371/journal.pone.0268472 (PMC9129035; doi:10.1371/journal.pone.0268472)

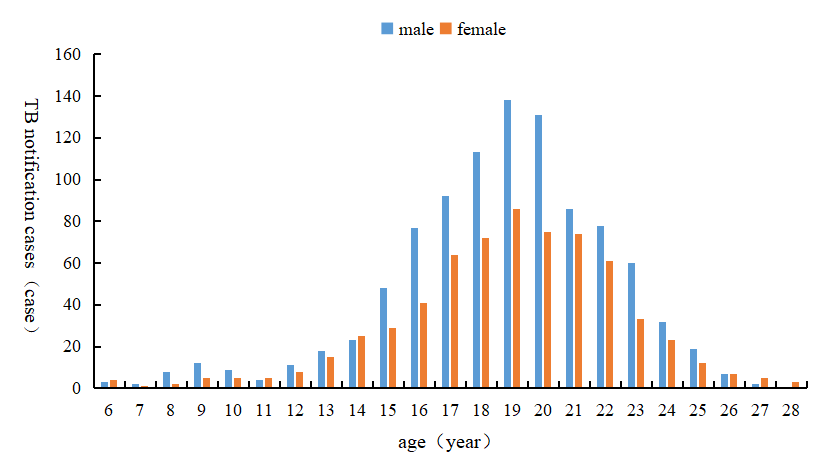

Supplement: S1 Fig — (PNG) [file pone.0268472.s001.png]

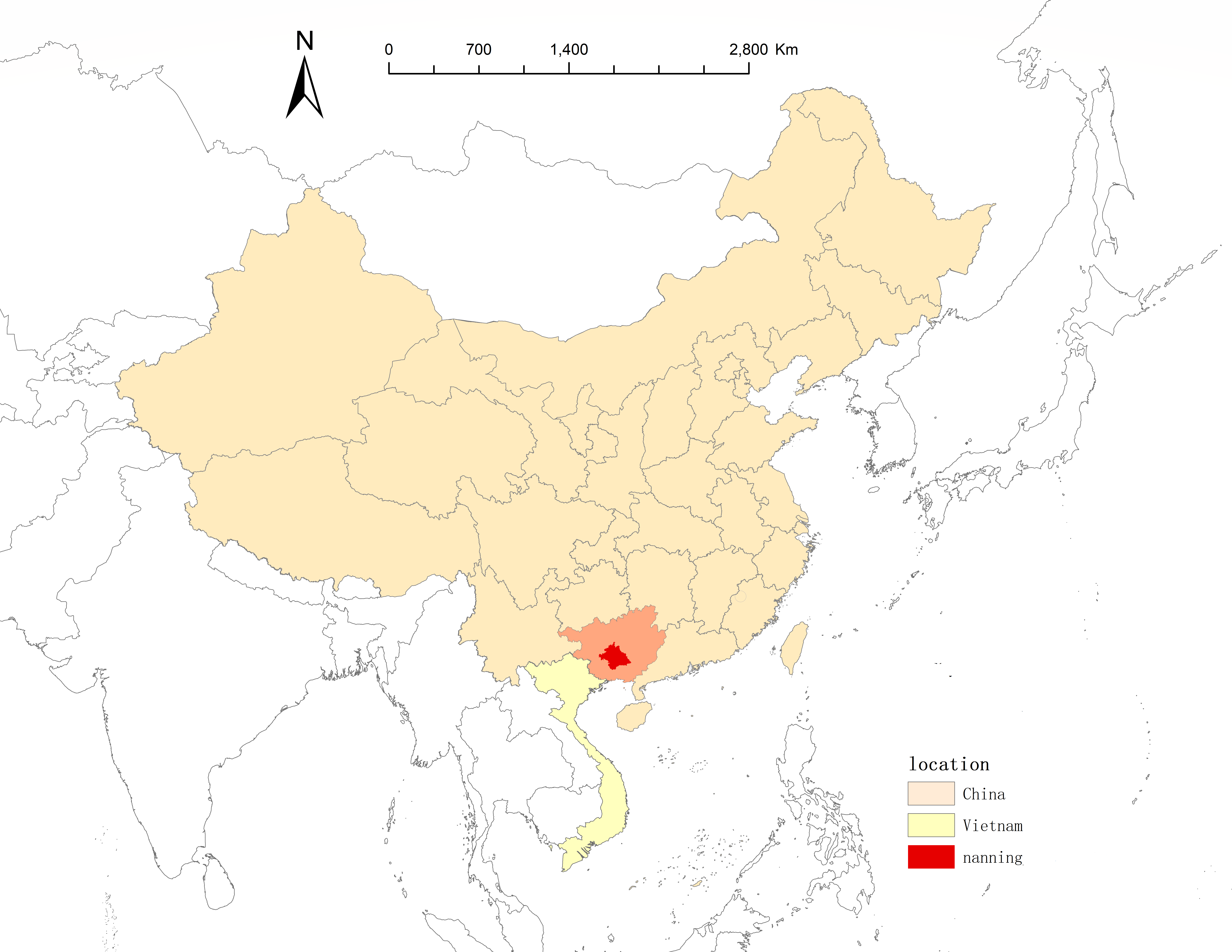

Supplement: S2 Fig — (PNG) [file pone.0268472.s002.png]
