# Supplementary material for: Spatial analysis and influencing factors of pulmonary tuberculosis among students in Nanning, during 2012–2018
Source: PLoS One. 2022 May 24;17(5):e0268472. doi: 10.1371/journal.pone.0268472 (PMC9129035; doi:10.1371/journal.pone.0268472)
Supplement: S1 Table — (DOCX) [file pone.0268472.s003.docx]

Local Indicators of Spatial Association (LISA) for annual TB notification rate among students in Nanning, from 2012 to 2018

| year | high-high cluster | high-low cluster | low-high cluster | low-low cluster | No Statistical   significant |
| --- | --- | --- | --- | --- | --- |
| 2012 | 6 | 5 | 4 | 0 | 96 |
| 2013 | 6 | 1 | 4 | 2 | 98 |
| 2014 | 7 | 1 | 3 | 0 | 100 |
| 2015 | 6 | 2 | 5 | 1 | 97 |
| 2016 | 8 | 3 | 3 | 3 | 94 |
| 2017 | 7 | 4 | 3 | 2 | 95 |
| 2018 | 8 | 3 | 3 | 5 | 92 |

Time trends of incidence rates of TB among students in Nanning, from 2012 to 2018

| year | AMPC | AMPC (95% CI) | *P* value |
| --- | --- | --- | --- |
| 2012 | 0.8 | -23.5-32.9 | >0.05 |
| 2013 | 2.3 | -18.0-27.5 | >0.05 |
| 2014 | 2.7 | -17.3-27.6 | >0.05 |
| 2015 | -1.1 | -30.7-41.1 | >0.05 |
| 2016 | 4.3 | -16.2-29.9 | >0.05 |
| 2017 | 8.1 | -29.3-65.4 | >0.05 |
| 2018 | -5.0 | -42.5-56.8 | >0.05 |

Related data

Reported incidence of pulmonary tuberculosis among students in Nanning, from 2012 to 2018

| year | 2012 | 2013 | 2014 | 2015 | 2016 | 2017 | 2018 |
| --- | --- | --- | --- | --- | --- | --- | --- |
| TB notification cases | 206 | 229 | 222 | 211 | 234 | 241 | 266 |
| TB notification rates | 12.82 | 14.46 | 13.79 | 13.08 | 16.28 | 17.57 | 15.86 |

Overall age distribution of reported cases of pulmonary tuberculosis among students in Nanning, from 2012 to 2018

| age | case | rate |
| --- | --- | --- |
| 6 | 7 | 1.05 |
| 7 | 3 | 0.45 |
| 8 | 10 | 1.51 |
| 9 | 17 | 2.56 |
| 10 | 14 | 2.22 |
| 11 | 9 | 1.43 |
| 12 | 19 | 3.01 |
| 13 | 33 | 5.23 |
| 14 | 48 | 7.61 |
| 15 | 74 | 10.79 |
| 16 | 115 | 16.77 |
| 17 | 152 | 22.16 |
| 18 | 184 | 26.83 |
| 19 | 224 | 32.66 |
| 20 | 204 | 26.47 |
| 21 | 159 | 20.63 |
| 22 | 136 | 17.64 |
| 23 | 92 | 11.94 |
| 24 | 55 | 7.14 |
| 25 | 30 | 4.10 |
| 26 | 14 | 1.91 |
| 27 | 7 | 0.96 |
| 28 | 3 | 0.41 |

Temporal distribution of reported cases of pulmonary tuberculosis cases by year among students in Nanning, from 2012 to 2018

|  | 2011 | 2012 | 2013 | 2014 | 2015 | 2016 | 2017 | 2018 | total |
| --- | --- | --- | --- | --- | --- | --- | --- | --- | --- |
| January | 2 | 7 | 18 | 18 | 24 | 17 | 15 | 20 | 121 |
| February | 0 | 28 | 19 | 15 | 9 | 9 | 9 | 14 | 103 |
| March | 1 | 26 | 37 | 23 | 16 | 30 | 29 | 37 | 199 |
| April | 0 | 20 | 15 | 22 | 14 | 22 | 27 | 26 | 146 |
| May | 2 | 28 | 24 | 32 | 21 | 22 | 15 | 20 | 164 |
| June | 2 | 20 | 13 | 15 | 12 | 18 | 14 | 11 | 105 |
| July | 3 | 10 | 13 | 7 | 16 | 16 | 12 | 18 | 95 |
| August | 0 | 10 | 12 | 11 | 9 | 14 | 16 | 16 | 88 |
| September | 0 | 12 | 20 | 24 | 27 | 22 | 35 | 44 | 184 |
| October | 2 | 19 | 23 | 17 | 23 | 26 | 23 | 21 | 154 |
| November | 1 | 13 | 15 | 17 | 25 | 22 | 20 | 30 | 143 |
| December | 6 | 13 | 20 | 21 | 15 | 16 | 26 | 9 | 126 |
| total | 19 | 206 | 229 | 222 | 211 | 234 | 241 | 266 | 1628 |
